# Development of Antithrombogenic ECM-Based Nanocomposite Heart Valve Leaflets

**DOI:** 10.1021/acsabm.2c00423

**Published:** 2022-07-15

**Authors:** Ahsen Seyrek, Gülçin Günal, Halil Murat Aydin

**Affiliations:** † Nanotechnology and Nanomedicine Division, Institute of Science, Hacettepe University, Beytepe, 06800, Ankara, Turkey; ‡ Bioengineering Division, Institute of Science, Hacettepe University, Beytepe, 06800, Ankara, Turkey; § Centre for Bioengineering, Hacettepe University, Beytepe, 06800, Ankara, Turkey

**Keywords:** Heart valve, Decellularization, Thrombogenicity, Pericardium, Carbon nanotube

## Abstract

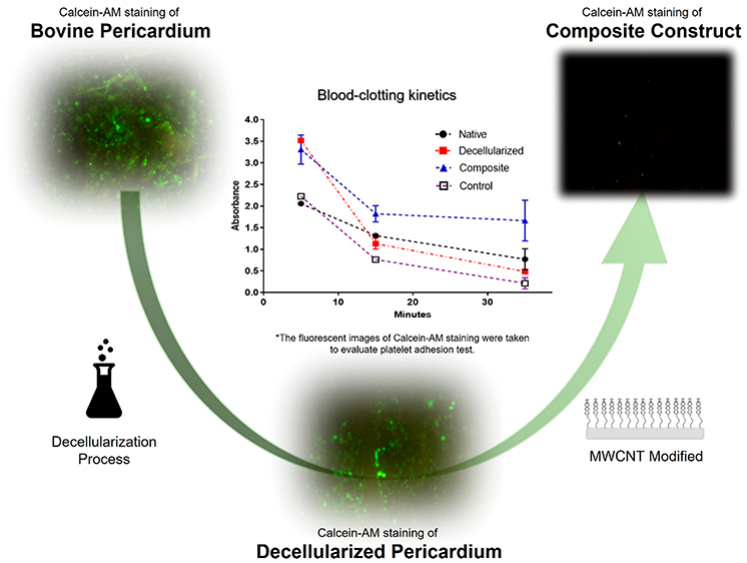

Thrombogenicity, which is commonly encountered in artificial heart valves after replacement surgeries, causes valvular failure. Even life-long anticoagulant drug use may not be sufficient to prevent thrombogenicity. In this study, it was aimed to develop a heart valve construct with antithrombogenic properties and suitable mechanical strength by combining multiwalled carbon nanotubes within a decellularized bovine pericardium. In this context, the decellularization process was performed by using the combination of freeze–thawing and sodium dodecyl sulfate (SDS). Evaluation of decellularization efficiency was determined by histology (Hematoxylin and Eosin, DAPI and Masson’s Trichrome) and biochemical (DNA, sGAG and collagen) analyses. After the decellularization process of the bovine pericardium, composite pericardial tissues were prepared by incorporating −COOH-modified multiwalled carbon nanotubes (MWCNTs). Characterization of MWCNT incorporation was performed by ATR-FTIR, TGA, and mechanical analysis, while SEM and AFM were used for morphological evaluations. Thrombogenicity assessments were studied by platelet adhesion test, Calcein-AM staining, kinetic blood clotting, hemolysis, and cytotoxicity analyses. As a result of this study, the composite pericardial material revealed improved mechanical and thermal stability and hemocompatibility in comparison to decellularized pericardium, without toxicity. Approximately 100% success is achieved in preventing platelet adhesion. In addition, kinetic blood-coagulation analysis demonstrated a low rate and slow coagulation kinetics, while the hemolysis index was below the permissible limit for biomaterials.

## 1 Introduction

Valvular heart disease (VHD) is currently one of the major cardiovascular problems that causes a significant rate of morbidity. Every year, more than 300,000 heart valve replacement surgeries are performed in the world for the treatment of VHD caused by various genetic or environmental reasons.^1^ Mechanical and bioprosthetic heart valves are used in these heart valve replacement surgeries. However, mechanical heart valves require lifelong use of anticoagulant medication due to thrombogenic and hemolytic complication problems.^2^ On the other hand, although it is less common than mechanical heart valves, long-term thrombosis can be seen in bioprosthetic heart valves as well.^3−7^ The thrombosis process is mediated with binding of certain plasma proteins on the surface of valves through a cascade of reactions, followed by platelet adhesion and activation, leading to thrombus formation.^8^ Properties such as hydrophilicity/hydrophobicity, surface charge and surface roughness regulate the interaction between platelet and biomaterial.^9^ By taking these parameters into consideration, development of antithrombogenic biomaterials for bioprosthetic heart valves is being widely studied.^10,11^ Natural polymers such as collagen, alginate, and chitosan; synthetic polymers such as poly(glycerol sebacate), poly(ethylene glycol), polycaprolactone, and poly-4-hydroxybutrate; and decellularized scaffolds such as bovine pericardium and porcine heart valve are used for bioprosthetic heart valves.^12,13^ An ideal heart valve material should provide mechanical and hemodynamic properties of natural heart valves. However, polymers cannot fully form the extracellular matrix structure in three dimensions and provide tissue hemodynamics,^14^ while decellularized scaffolds mimic the three-dimensional structure of the extracellular matrix at the nanoscale and show better hemodynamic and mechanical properties compatible with leaflets. Decellularization is performed in order to minimize the risk of immune response in tissues, by removing the cells and antigens. Physical methods (freezing-thawing, sonication), chemical agents (detergent, acidic and alkaline solutions), and enzymatic agents (nuclease, calcium chelating agent) are used for this procedure. However, decellularization processes damage the extracellular matrix structure of the tissue and reduce its mechanical strength.^15,16^

Bovine pericardium is the most commonly used material in bioprosthetic heart valves. It has advantages over synthetic materials such as unlimited supply of donor tissue, lower cost, suitable mechanical strength, functional host tissue integration, greater biocompatibility and hemodynamic profile, and lower infection risk. In addition, the fact that the rate of degeneration and reoperation is lower than that for the porcine heart valve makes bovine pericardium prominent in studies.^17^ However, the thrombogenicity-promoting feature of collagen in decellularized pericardial tissue necessitates the application of surface modification to the tissue.^18,19^

When it is desired to improve the properties of decellularized tissues for various purposes, in order to mimic the three-dimensional nanoscale structure of the extracellular matrix in the best way, decellularized tissues can be incorporated with nanomaterials. Nanocomposite scaffolds have many advantages besides structural similarity to the extracellular matrix, such as high surface area to volume ratio, and adjustability in surface functionality and mechanical properties.^20,21^

Carbon nanotubes (CNTs) are made of rolled graphite sheets with nanoscale diameters. CNTs have unique chemical, electrical, and mechanical properties. They provide high aspect ratio due to its nanoscale diameters and micrometer lengths. Due to this property, CNTs are preferred for biomedical applications as they can efficiently interact with cells and tissues. In addition, its high aspect ratio provides the flexibility required for use in the heart valve materials, and its inert structure minimizes the risk of any reaction. Furthermore, high blood compatibility and antithrombogenicity properties of carbon nanotube modified synthetic and natural polymers have been reported in the literature. In these studies, it was indicated that modification with CNTs showed a low hemolysis index and greatly reduced platelet adhesion and activation in the biomaterial surfaces.^22−25^

In this study, a nanocomposite heart valve material was developed to prevent thrombogenicity that may occur with artificial heart valves. Decellularized bovine pericardium, which is frequently used in bioprosthetic heart valves, was incorporated with −COOH modified multiwalled carbon nanotubes, known with antithrombogenic properties and compatible nanostructure with extracellular matrix.

## 2 Materials and Methods

### 2.1 Materials

Sodium dodecyl sulfate (SDS) and hematoxylin were purchased from Merck, Germany. Tris-HCl, Eosin, Proteinase K, chondroitin sulfate, collagenase Type IA, and phosphate buffered saline (PBS) were purchased from Sigma-Aldrich, USA. Masson Trichrome was purchased from Scytek, USA and DAPI was purchased from Biotium, USA. Quant-iT PicoGreen assay was purchased from Invitrogen, USA, and hydroxyproline assay was purchased from Biovision, USA. −COOH functionalized multiwalled carbon nanotube (Purity: >96%, Outside Diameter: 28–48 nm) was supplied from Nanografi, Turkey. Sheep blood was obtained from the slaughterhouse. For cytotoxicity studies, L929 Mouse Fibroblast cell line (ATCC, USA) was purchased. Low glucose Dulbecco’s medium (L-DMEM), antibiotic–antimycotic solution, fetal bovine serum (FBS), l-glutamine, Trypsin-EDTA (0.25%-w/) PhenolRed) was purchased from Capricorn, Germany. The MTT (3-(4,5-dimethylthiazol-2-yl)-2,5-diphenyltetrazolium bromide) assay kit was purchased from Biovision, USA.

### 2.2 Preparation and Characterization of the Decellularized Bovine Pericardium

Bovine hearts obtained from the slaughterhouse were transported to the laboratory in 1 h and stored at −80 °C until use. The pericardial layer was dissected from the outermost surface of the hearts using a scalpel. The adherent fat tissue was gently removed from the pericardial surface. Using different chemical and mechanical methods, a decellularization protocol was optimized. Briefly, pericardial tissues were freeze–thawed in 10 mM hypotonic Tris-HCl buffer (pH = 7.4) at −80 and 25 °C following the treatment of tissues with 0.5% SDS (w/v) in an orbital shaker at room temperature for 24 h. Samples were washed with PBS for 48 h to remove residual DNA. The decellularization process was evaluated by histological and biochemical analysis. Native and decellularized samples were fixed with 10% formalin for 5 days. Samples were washed with PBS for removal of the fixative agent. After treatment with ethanol series and xylene, samples were embedded in paraffin and cut into 5 μm sections. Hematoxylin and eosin (H&E), DAPI, and Masson’s Trichrome stains were performed for histological analysis. Biochemical analyses were performed to determine residual amounts of DNA, sGAG, and collagen. Samples were lyophilized for 16 h (Labconco, USA) and enzymatically digested by treatment with 1 mg/mL solution of proteinase K in ammonium acetate for 16 h. Residual DNA was quantified with Quant-iT PicoGreen dsDNA Assay Kit. Absorbance values were measured by a fluorescence spectrophotometer (Agilent Cary Eclipse, USA) at an excitation wavelength of 480 nm and emission wavelength of 520 nm. Quantification of sGAG was determined by using dimethyl methylene blue assay (DMMB). Absorbance values were measured with microplate spectrophotometer (Epoch-BioTek, USA) at 525 nm. For the measurement of the collagen content, samples were treated with HCl for 3 h. After incubation with hydroxyproline assay reagents, absorbance values were measured at 560 nm. The obtained values were normalized according to 10 mg dry weight of samples.

### 2.3 Preparation of the MWCNT-Pericardium Composites

Composite constructs were obtained by incorporation of −COOH modified MWCNTs with decellularized pericardial matrices. Briefly, 0.05% −COOH modified MWCNT solution was prepared in deionized water, and homogeneous dispersion was formed by sonicating the solution for 2 h. After sonication, the COOH–MWCNT solution was incubated with decellularized pericardial tissues for 24 h, at 37 °C, for 120 rpm in an orbital shaker. At the end of this period, the tissues were removed from the solution and washed with PBS for 48 h in the orbital shaker to remove non-interacting carbon nanotubes. Finally, the samples were lyophilized for further studies.

### 2.4 Characterization of the MWCNT-Pericardium Composites

Chemical compositions of the native, decellularized, and composite samples were determined by attenuated total reflectance–Fourier transform infrared spectroscopy (ATR-FTIR) (Agilent, USA) in the wavenumber region between 400 and 4000 cm^–1^.

Carbon nanotube content in the composite pericardium was determined using a thermogravimetric analyzer (TA Instruments, SDT Q600, USA). The analysis was carried out at a constant heating rate of 10 °C/min, in an air atmosphere and temperature range of 0–900 °C.

Morphology of native, decellularized, and composite materials was examined by scanning electron microscopy (SEM) (Tescan, Czechia). Lyophilized samples were coated with gold–palladium (Au–Pd) for imaging in different magnifications.

In order to investigate the amount and distribution of carbon nanotubes interacting with the pericardial surface, native, decellularized, and composite scaffolds were analyzed by atomic force microscopy (AFM) (Oxford Instruments, England). Two- and three-dimensional AFM images were obtained in noncontact mode with a spring constant of 48 N/m, 190 kHz resonance frequency, and a tip diameter of 15 nm. Image processing was performed with Gwyddion software (Czech Republic).

Mechanical analyses of the native, decellularized, and composite constructs were performed using Univert Biomaterial Tester (CellScale, Canada) with a 50 N load cell at a 10 mm/min strain rate. The samples (1 × 1 cm^2^, *n* = 3) were equilibrated in PBS for 1 h. Tensile strength and Young’s moduli values, calculated from the 10–40% strain range in the stress–strain curve, were obtained.

The hydrophilicity/hydrophobicity of native, decellularized, and composite pericardial surfaces was evaluated by contact angle measurement (Biolin Scientific, Attension Theta). 1 μL of distilled water was dropped onto each surface, and contact angles between droplet and surface were measured. The contact angle data were obtained from two different positions of the samples. Results were expressed as mean ± standard deviation. In addition to contact angle measurement, surface energies of native, decellularized, and composite pericardial constructs were calculated using the Young-Dupré equation, and it is expressed in eq 1.^26^
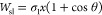
1where *W*_sl_ is the surface energy of solid (mJ/m^2^); σ_1_ is the surface tension of liquid (mJ/m^2^), and θ is contact angle (deg).

In order to evaluate in vitro stability of native, decellularized, and composite constructs, all groups degraded enzymatically for 24 h. At first, all samples (*n* = 3) were lyophilized, and the initial weights (*W*_0_) were determined. The samples were treated in collagenase solution (2 U/mg tissue) in 100 mM PBS at pH 7.5 for 24 h. The experiment was carried out in a Thermoshaker (Gerhardt, Germany) and incubated at 37 °C and 30 rpm. To determine the final weights (*W*_f_), all samples were washed with 100 mM PBS and lyophilized for 16 h. The degradation rate was determined by the following equation:
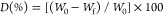
2where *W*_0_ is the initial dry weight and *W*_f_ is the dry weight after degradation.

### 2.5 Platelet Adhesion Test

Platelet adhesion testing was carried out according to ISO 10993-4 standards. Platelet adhesion, aggregation, and activation response of native, decellularized, and composite scaffolds were investigated in thrombogenicity studies. Briefly, anticoagulated sheep blood obtained from the slaughterhouse was quickly delivered to the laboratory in blood tubes mixed with 3.8% (w/v) sodium citrate solution at a ratio of 9:1. Anticoagulated blood was centrifuged at 1700 rpm for 10 min to obtain platelet-rich plasma (PRP). Platelet concentration in the plasma was determined to be approximately 2 × 10^8^/mL. 500 μL of platelet-rich plasma was added to the samples placed in a 24-well plate and incubated at 37 °C, 100 rpm in an orbital shaker for 1 h. The samples were washed with PBS twice to remove nonadherent platelets followed by the samples being fixed with 2.5% (v/v) glutaraldehyde/PBS solution at 4 °C. After the samples were rinsed with PBS, they were dehydrated with a series of graded alcohol solutions (0%, 25%, 50%, 75%, and 100% (v/v)) for 10 min. Finally, the samples were lyophilized, coated with gold–palladium (Au–Pd), and examined by SEM at various magnifications (Tescan, Czechia).

In order to confirm the SEM images and quantify the amount of cell adhesion and viability more accurately on the surfaces of native, decellularized, and composite pericardium that interact with platelet-rich plasma, Calcein-AM staining was also performed. In this staining, living cells are stained green due to intracellular esterase activity and fluorescence. 500 μL of platelet-rich plasma was added to the 1 × 1 cm^2^ samples placed in a 24-well plate and incubated for 1 h at 100 rpm in an orbital shaker at 37 °C. At the end of the incubation, samples were washed with PBS twice to remove nonadherent platelets followed by treatment with 500 μL of Calcein-AM dye (2 μM) and incubation for 30 min. Afterward, the materials were washed twice with PBS and examined under a green filter with a fluorescence microscope (Leica, Germany).

### 2.6 Kinetic Blood-Clotting Time Assay

Kinetic blood-clotting time experiments were performed to examine the coagulation reaction that occurs when samples interact with blood. Clotting dynamics of the blood on the surfaces was investigated after 5, 15, and 35 min of interaction. Three samples from native, decellularized, and composite pericardium were prepared at 1 × 1 cm^2^ and placed in a 12-well plate. First, activated blood was prepared by interacting 3 mL of anticoagulated blood with 300 μL of 0.1 M CaCl_2_. 100 μL of activated blood was added to the samples, and they were incubated at room temperature for 5, 15, and 35 min. At the end of each time slot, the samples were incubated with 2 mL of distilled water for 5 min. By the addition of distilled water, red blood cells that are not trapped in the thrombus are lysed, and hemoglobin is released into the solution. 200 μL of solution from each sample was transferred to a 96-well plate, and free hemoglobin concentration in the solution was measured at 540 nm with spectrophotometer. The absorbance values of the solutions vs time were plotted. Blood added to the empty well was used as a positive control.

### 2.7 Hemolysis Assay

Evaluation of red blood cell disruption on native, decellularized, and composite pericardial surfaces were studied by hemolysis analysis. Anticoagulated blood was diluted by mixing with PBS at a ratio of 4:5. Samples (1 × 1 cm^2^) were placed in a centrifuge tube and incubated with 10 mL of PBS at 37 °C for 30 min; following that, 200 μL of diluted blood was added to the samples and incubated at 37 °C. After 1 h, the solutions in the tubes were centrifuged at 1500 rpm for 10 min. 100 μL of the supernatants was transferred to a 96-well plate, and absorbance values were read at 540 nm by using a spectrophotometer. Diluted blood with PBS was taken as the negative control, while diluted blood with distilled water was the positive control. The hemolytic rate was calculated with the following formula:
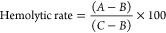
3where *A*, *B*, and *C* are the absorbance values of the samples, negative control, and positive control, respectively.

### 2.8 In Vitro Cytotoxicity Assay

In order to examine the effects of composite pericardium on cell attachment, growth, and proliferation, MTT (3- [4,5-dimethylthiazol-2-il]-diphenyltetrazolium bromide) analysis was performed. Lyophilized samples (*n* = 3) were prepared at 1 × 1 cm^2^ and kept in 70% (v/v) ethanol for 20 min for sterilization, and then treated with 3% AA (Antibiotic–Antimycotic solution dissolved in PBS) for 45 min. After rinsing with PBS, the samples were air-dried, and UV sterilized. 1% l-glutamine, 1% Antibiotic–Antimycotic, and 10% fetal bovine serum (FBS) were added to low glucose DMEM medium. L929 cells were seeded into the 24-well plate at a 4.5 × 10^4^ cells/well density. Cells seeded into an empty well were taken as the control group. Samples were kept in the incubator for 3 h, and then 1 mL of medium was added.

After 24 h of incubation, 2.5 mg/mL MTT solution was prepared in PBS. The medium in the wells was removed, and 600 μL of DMEM and 60 μL of MTT solution were added onto the samples. After samples were kept in a CO_2_ incubator for 3 h, the solution on them was removed and the formazan crystals formed were dissolved by adding 400 μL of DMSO. 200 μL aliquots of solutions were taken from each well, placed in a 96-well plate, and the absorbance values measured in a microplate reader at 570 nm. 690 nm was taken as the reference absorbance value.

### 2.9 Statistical Analysis

The data obtained from the analyses were expressed as mean ± standard deviation. For multiple statistical comparisons, the one-way ANOVA method was used, while Student’s *t* test was performed in the comparison of two groups. Values of *p* < 0.05 were considered significantly different.

## 3 Results

### 3.1 Evaluation of Decellularization Process

**Figure 1:**
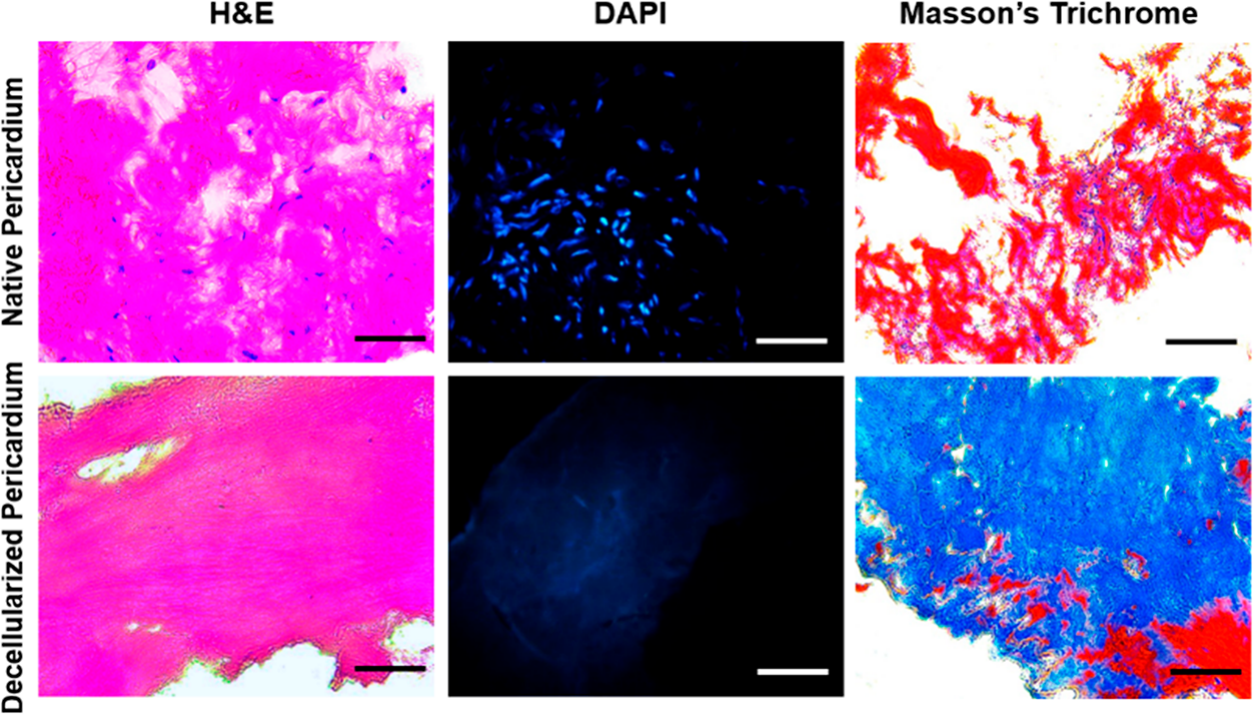
Haematoxylin & Eosin, DAPI, and Masson’s Trichrome stainings of native and decellularized pericardium (scale bar: 800 μm).

The efficiency of the decellularization process was evaluated by histological and biochemical analysis. While cell nuclei and extracellular matrix integrity were examined with Haematoxylin & Eosin (H&E) and DAPI staining, distribution of collagen in the extracellular matrix was characterized by Mason’s Trichrome staining (Figure 1). No obvious residual DNA was observed in H&E and DAPI stainings of decellularized bovine pericardium. In addition, ECM structure and integrity were found to be preserved in H&E and Masson’s Trichrome stainings. Blue-stained collagen fibers were found to be increased in decellularized pericardium in comparison with native pericardium, as the decellularization process revealed more collagen fibers due to detrimental effects of decellularization process.

**Figure 2:**
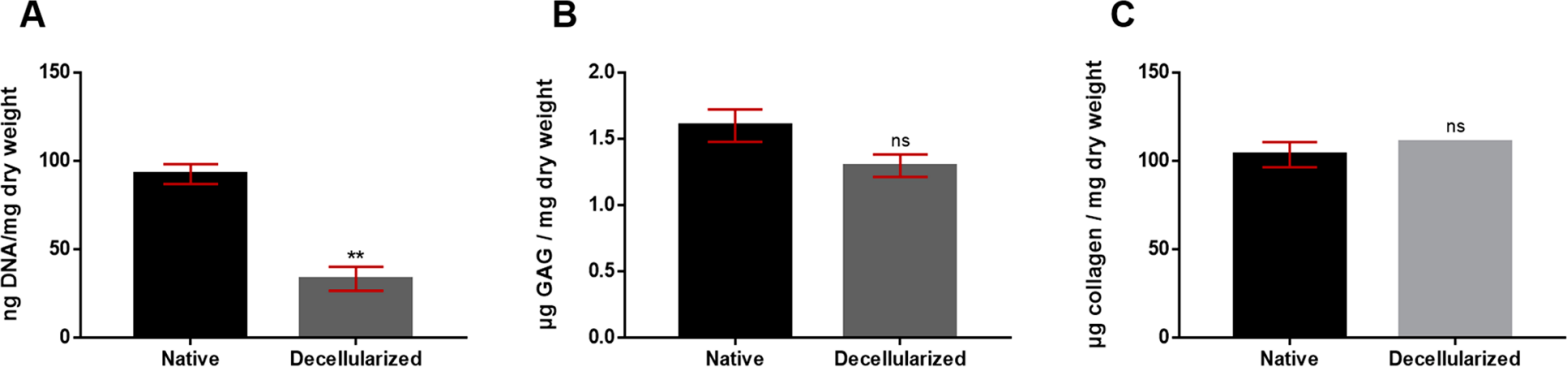
Biochemical analysis of the amount of residual DNA (A), sulfated-glycosaminoglycan (sGAG) (B), and collagen (C) of native and decellularized pericardium (**p < 0.01, *n* = 3).

Quantitatively, the amount of residual DNA was decreased from 92.41 ± 3.96 ng DNA/mg dry weight to 33.15 ± 5.51 ng DNA/mg dry weight, as can be seen from Figure 2A. While the sGAG content in the native pericardium was found to be 1.60 ± 0.07 μg sGAG/mg dry weight, it was 1.30 ± 0.06 μg sGAG/mg dry weight in the decellularized pericardium (Figure 2B). The collagen level in native and decellularized pericardium indicated no significant difference. 103.4 ± 4.098 μg and 110.4 ± 0.0093 μg was found in native and decellularized pericardium, respectively (Figure 2C). These results were also found to be compatible with Masson’s Trichrome staining. Statistically, a significant difference was observed only in the DNA content of the native and decellularized groups (**p < 0.01).

### 3.2 Characterization of Decellularized Pericardium and MWCNT-Pericardium Composites

#### 3.2.1 Attenuated Total Reflectance–Fourier Transform Infrared Spectroscopy (ATR-FTIR)

**Figure 3:**
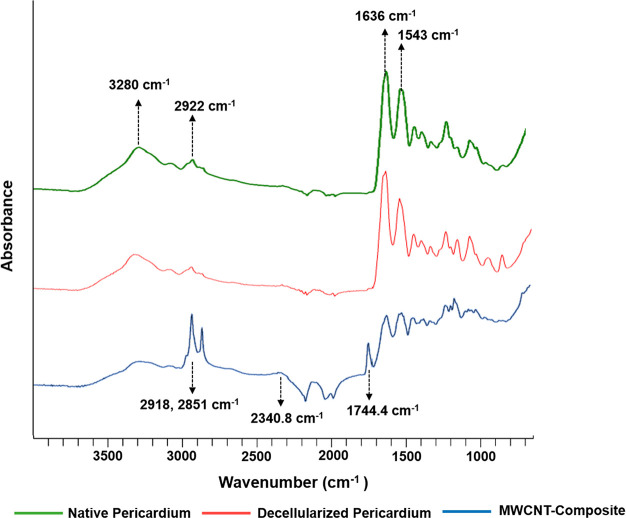
ATR-FTIR spectrum of native, decellularized, and composite pericardium.

Surface characterization of native, decellularized, and composite constructs were performed by ATR-FTIR analysis (Figure 3). In native and decellularized pericardium spectra, the characteristic peak at 1636.3 cm^–1^ was attributed to amid-I, and the peak at 1543.1 cm^–1^ represents amide-II bonds due to N–H bending vibration. Moreover, the peak at 2922.2 cm^–1^ wavelength indicates the C–H groups found in glycosaminoglycans, while the peaks between 3280.1 and 3309.9 cm^–1^ demonstrates the presence of amide-A. In the composite pericardium, apart from the FTIR spectrum of the decellularized pericardium, the peak at 3272.6 cm^–1^ corresponds to the hydroxyl (O–H) groups of carboxyl groups in carbon nanotubes, the density increases at 2918.5 cm^–1^, and the newly formed peak at 2851.4 cm^–1^ were ascribed to the C–H bonds of H–C=O in carbonyl group. Furthermore, the peak in the 1740 cm^–1^ shows the C=O bond in the −COOH groups of the carbon nanotubes, and the peak appearing in the 2360 cm^–1^ belongs to the O–H bond in the strongly hydrogen-bonded −COOH groups.^27,28^

#### 3.2.2 Thermal Gravimetric Analysis (TGA)

**Figure 4:**
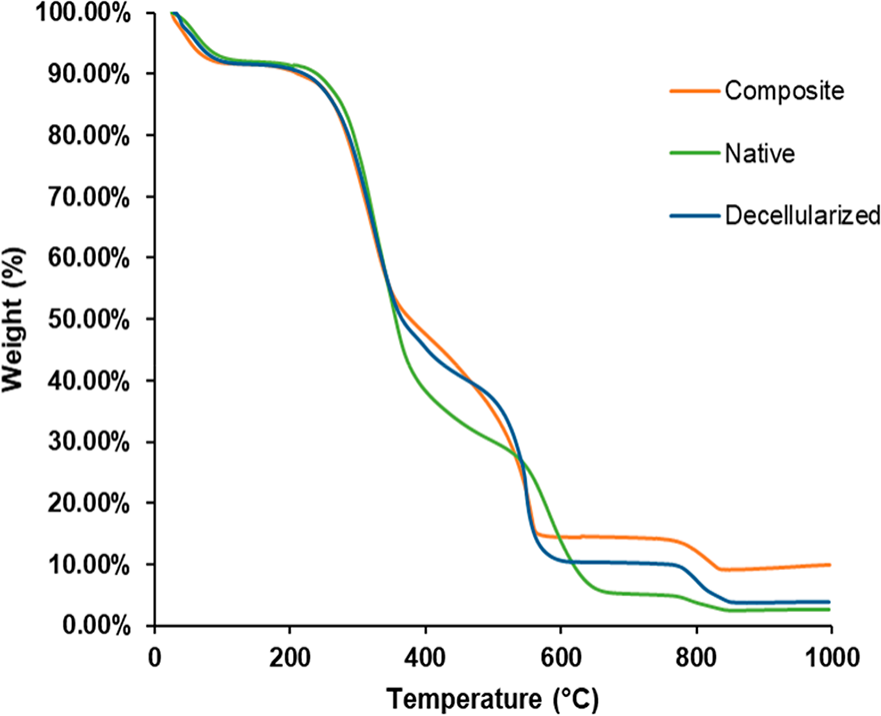
Thermogravimetric analysis of native, decellularized, and composite pericardium.

In order to determine the amount of carbon nanotubes interacting with the decellularized pericardium, thermogravimetric analysis was carried out on native, decellularized, and composite constructs (Figure 4). Weight changes up to about 200 °C indicate adsorbed water and moisture in the tissue, while the range of 200–400 °C indicates extracellular matrix loss. As can be found from Figure 4, the amount of water and moisture in the tissues was found to be around 10%, while the extracellular matrix components (90% collagen) constitute about 50% of the tissue. The second sharp weight loss between 450 and 600 °C is the region where inorganic components are decomposed.^28,29^ It was found that the thermal stability of carbon nanotubes started to decrease after 400 °C. When the residual weights were compared, no significant difference was found between native and decellularized pericardium. Nevertheless, the amount of inorganic components was significantly higher in the composite than the native and decellularized pericardium after 600 °C. A 9.74% (w/w) inorganic component was found in the composite pericardium, while these were 2.64% and 3.85% (w/w) in native and decellularized pericardium, respectively.

#### 3.2.3 Scanning Electron Microscopy (SEM)

**Figure 5:**
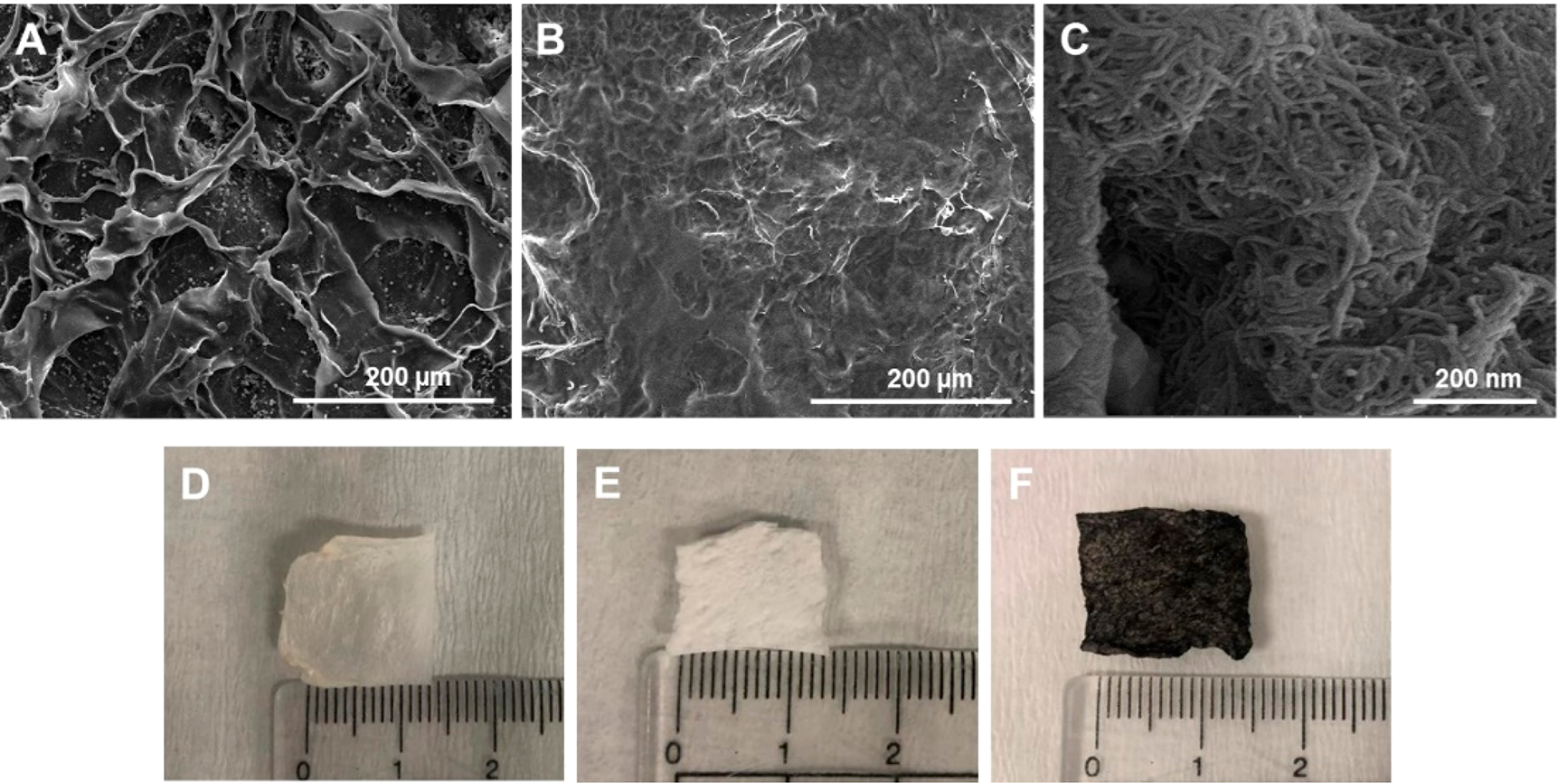
SEM and macroscopic images of native (A, D), decellularized (B, E), and composite pericardium (C, F).

Surface morphologies of native, decellularized, and composite constructs were analyzed by scanning electron microscopy. SEM images (Figure 5) demonstrated that while native pericardium has a more stratified and heterogeneous morphology, this structure became flatter and smoother after the decellularization process (Figure 5A,B). Ultimately, the decellularized pericardium was found to retain its matrix integrity. Figure 5C, on the other hand, proved the interaction and homogeneous distribution of carbon nanotubes on the pericardial surface. In addition to microscopic images obtained from SEM, macroscopic images of native, decellularized, and composite pericardium were given in Figure 5D, E, and F, respectively.

#### 3.2.4 Atomic Force Microscopy (AFM)

**Figure 6:**
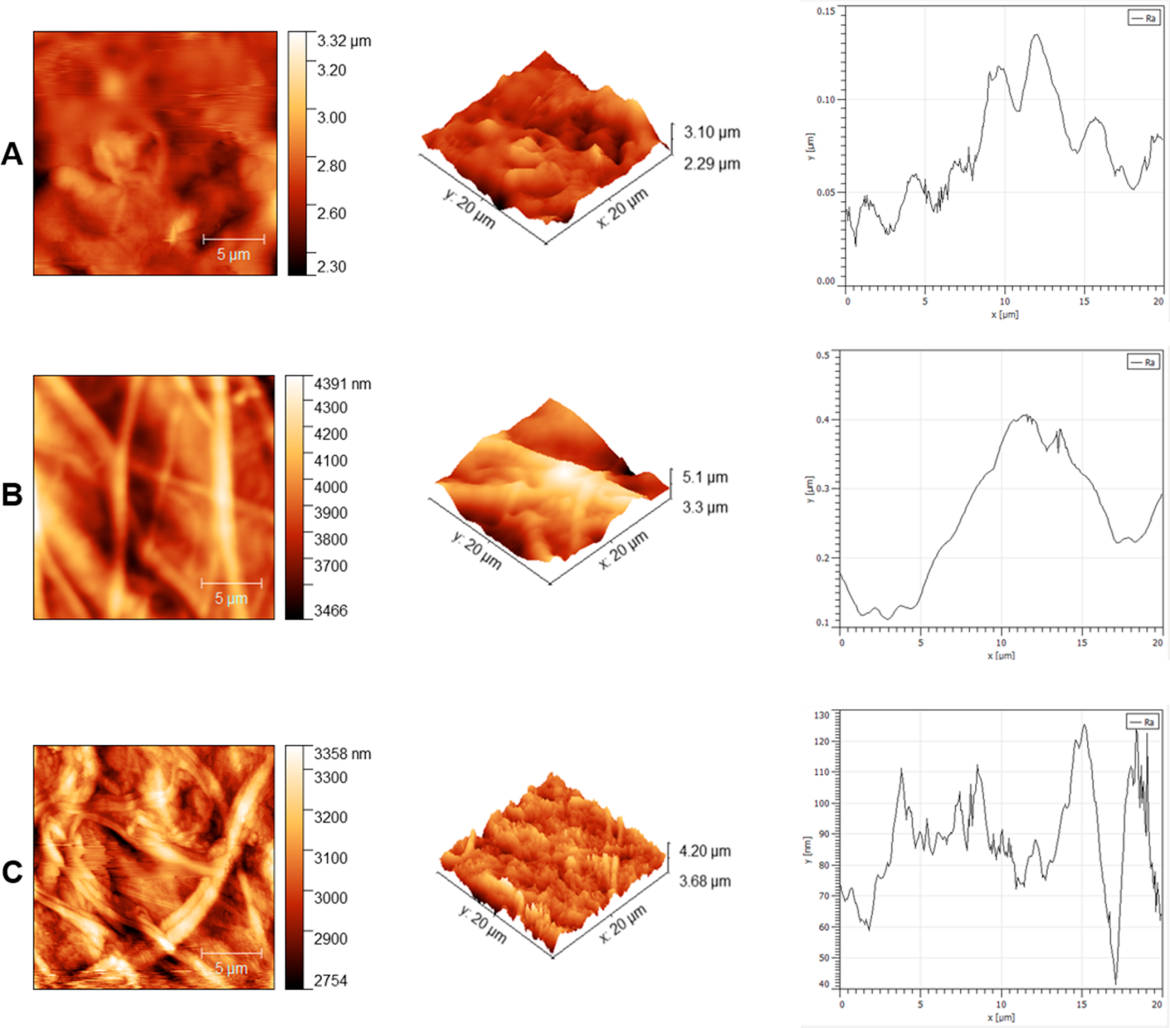
AFM images of native (A), decellularized (B), and composite (C) pericardium.

Native, decellularized, and composite pericardium were characterized by AFM in order to examine the efficiency of the carbon nanotube–pericardium interaction and distribution at the nanolevel. Phase images and roughness graphics were presented in Figure 6. While residual fat tissue and various surface roughness were observed on the native pericardium surface, it was observed that the roughness decreased, and a flatter surface was formed in the decellularized pericardium. Differences in peak intensity between graphs also support the phase images. It was also seen that collagen fibers mostly lie parallel to each other or were aligned in a certain gap and overlap phase. In Figure 6C, interaction and homogeneous distribution of carbon nanotubes on the pericardium surface were observed from phase images and the roughness graph. Significantly more sharp peaks were observed in the composite than the native and decellularized pericardium. In other words, the roughness was increased by the interaction of MWCNT-pericardium.

#### 3.2.5 Mechanical Evaluation

**Figure 7:**
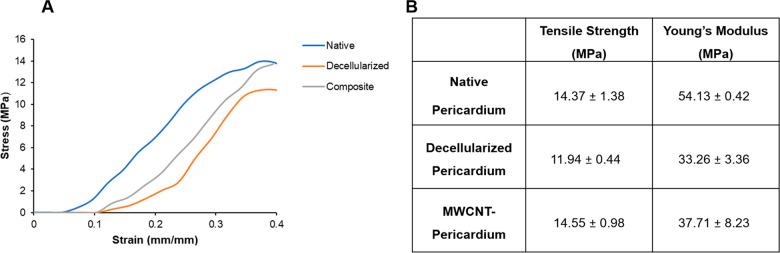
Stress–strain curve (A), tensile strength, and Young’s modulus data of native, decellularized, and composite pericardium (B).

The mechanical properties of native, decellularized, and composite pericardium were obtained to evaluate the effects of surface modification with carbon nanotubes. The stress–strain curves of the pericardial constructs are given in Figure 7. Young’s modulus, calculated by the slope over the 10–40% deformation area, was found to be the highest in the native pericardium with a value of 54.13 ± 0.42 MPa. While a decrease was observed after decellularization process (33.26 ± 3.36 MPa), composite pericardium was found to be 37.71 ± 8.23 MPa, demonstrating that modification with carbon nanotubes did not make a significant difference in terms of elastic modulus when compared to the decellularized pericardium.

Tensile strength, on the other hand, increased with carbon nanotube modification of the pericardial surface. Tensile strength of the native pericardium, found as 14.37 ± 1.38 MPa, decreased to 11.94 ± 0.44 MPa with the decellularization process. After modification with carbon nanotubes, tensile strength of the decellularized pericardium increased to 14.55 ± 0.98 MPa, indicating that it restored the tensile strength of native pericardium.

#### 3.2.6 Contact Angle Measurement

**Figure 8:**
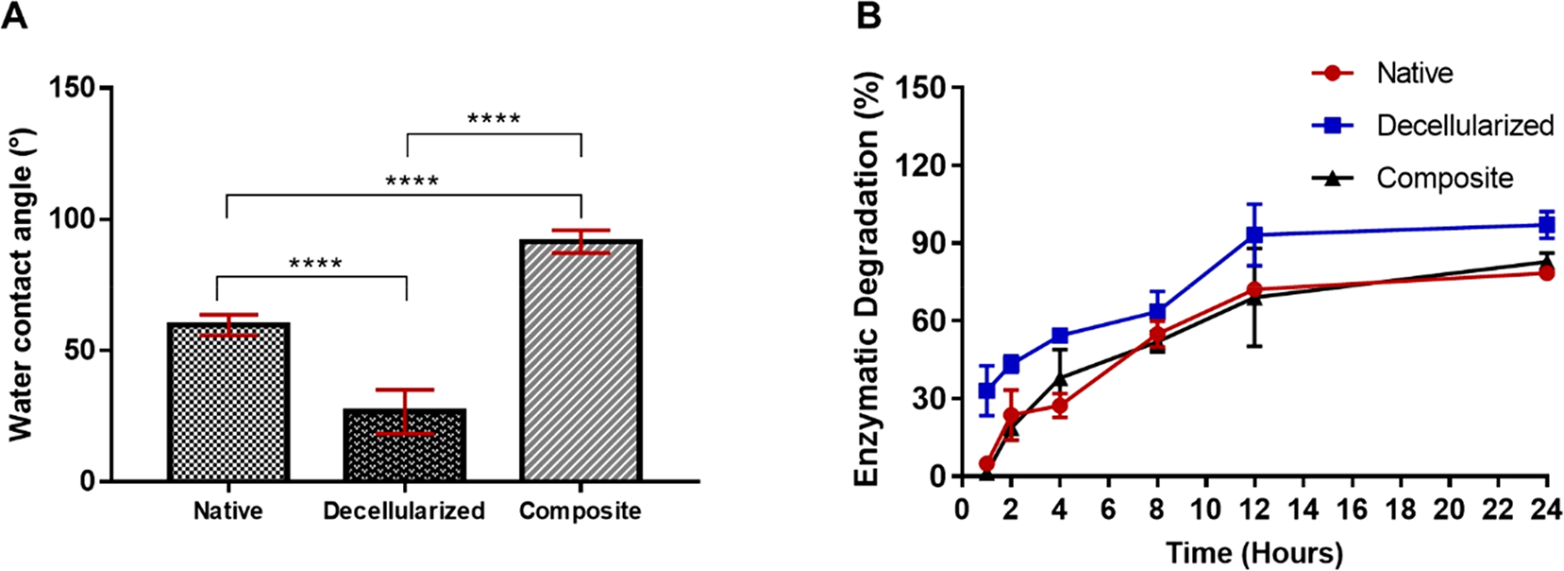
Contact angle measurement of native, decellularized, and composite pericardium (A), enzymatic degradation profiles of native, decellularized, and composite constructs in collagenase solution (B) (*n* = 3, ****p < 0.0001).

The results of contact angle measurements were shown in Figure 8A. The contact angle of native pericardium (59.66 ± 3.93°) was greatly decreased after the decellularization process found as 26.70 ± 8.12° due to the exposure of collagen tissue. On the other hand, carbon nanotube modification increased the contact angle of the decellularized pericardium to 91.50 ± 4.29° by providing the most hydrophobic nature among all. In addition, surface energies of native pericardium, decellularized pericardium, and composite construct were calculated as 109.57 ± 5.1 mJ/m^2^, 137.8 ± 8.2 mJ/m^2^, and 70.9 ± 3.5 mJ/m^2^, respectively, according to the Young–Dupré eq 1. Statistically significant differences were found among all groups (***p < 0.001). Compatible with the contact angle measurements, the surface energy was found to increase because of the decrease in surface hydrophobicity after the decellularization process of the native tissue. In addition, a decrease in surface energy was observed due to the increase in surface hydrophobicity with carbon nanotube modification.

#### 3.2.7 In Vitro Degradation Analysis

On behalf of a residual amount of degraded matrices after 24 h in collagenase solution, degradation profiles of each group were given in Figure 8B. Accordingly, while the degradation rate of native tissue was 4.9 ± 2.6% at the end of 1 h, this ratio was determined as 78.3 ± 2.1% at the end of 24 h. While the degradation ratio of decellularized matrices after 1 h treatment was 33 ± 9.7%, this ratio was 97.1 ± 5.1% at the end of 24 h. Moreover, the degradation ratio of composite structures after 1 h treatment was determined as 1.4 ± 0.1% and 82.8% ± 3.4% at the end of 24 h. It is obviously seen from the degradation profiles that the stability of the decellularized tissue is lower than that of the native tissue and composite structure. On the other hand, the composite structure shows a similar profile as native tissue.

### 3.3 Platelet Adhesion Test

**Figure 9:**
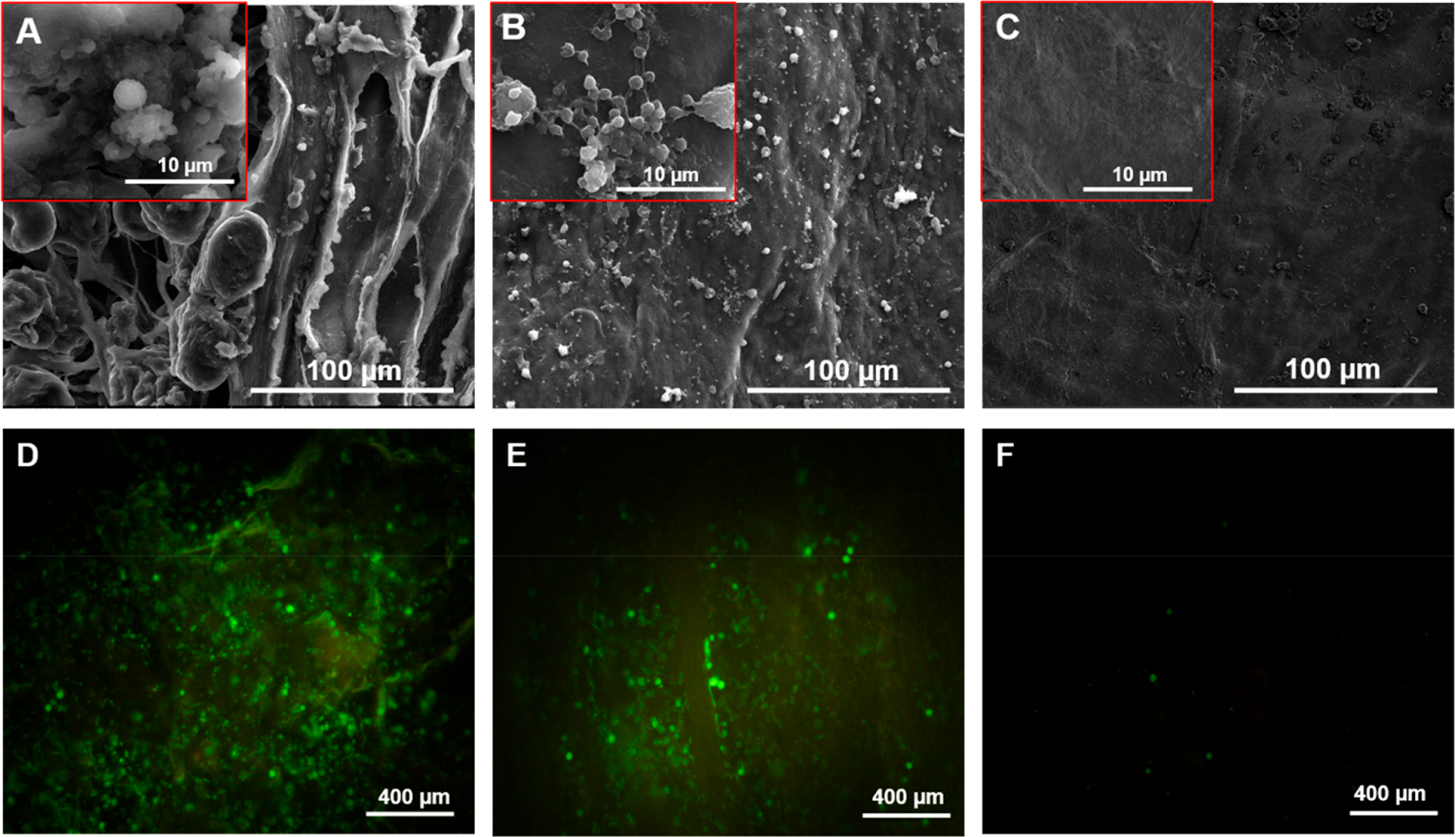
SEM and Calcein-AM images of platelets adhered on native (A, D), decellularized (B, E), and composite (C, F) pericardium.

Platelet adhesion testing, which is the one of the most important characterization methods for evaluating the thrombogenicity properties of materials, was carried out according to the ISO 10993-4 standard. SEM images of surfaces interacting with platelets are given in Figure 9. High numbers of platelet adhesion were observed in both native and decellularized pericardium. As can be seen from Figure 9A–C, most of the surfaces were covered with aggregate platelets, while the number of single platelets was found to be very low. Red and white blood cells, which are found in small numbers in PRP, were also adhered to the native and decellularized surfaces together with platelets (Figure 9C). At 10 μm magnification, interconnected cell aggregates were observed indicating that platelets were not only adhered but also activated. However, in the composite pericardial surface, nearly 100% success was achieved in the prevention of platelet adhesion. While no cells were observed in Figure 9D, a few single platelets were observed in higher magnification (Figure 9E) showing round morphologies. The round morphology of these platelets indicates that they are in the passive state. Images were obtained from different sections of the surfaces (n = 3) at each magnification and average of the images were considered in the evaluation.

Platelet adhesion testing was also performed with Calcein-AM staining in order to characterize the number and viability of platelets interacting with the surfaces more precisely. The images of the stained surfaces examined under a fluorescent microscope were given in Figure 9D–F. While a high number of viable platelets were observed in native and decellularized surfaces, this number was found to be significantly lower in the microscope images of composite pericardium.

Calcein-AM staining images confirm the SEM results and proved that collagen structures in native and decellularized pericardium promote cell adhesion, while modification with carbon nanotubes is effective in minimizing platelet adhesion and activation.

### 3.4 Hemolysis Assay

The hemolysis analysis, an indirect marker of thrombogenicity, was performed to investigate the interaction of the native, decellularized, and composite pericardium with red blood cells, and the groups were compared by calculating the hemolytic rate. Optical density at 540 nm and hemolysis rates are given in Table 1. While the hemolysis rate was found to be 1.42% in native pericardium, it was found to be 3.14 ± 0.006% in the decellularized pericardium. After the carbon nanotube modification, this value decreased to 1.07 ± 0.001% showing the good hemocompatibility properties of carbon nanotubes. Although decellularized pericardium has a higher hemolysis rate than the native and composite pericardium, all the materials were below 5%, which is the permissible limit for medical devices.^23^

**Table 1:** Optical Density and Hemolysis Index for Positive Control, Negative Control, Native, Decellularized, And Composite Pericardium

Samples	Optical density at 540 nm	Hemolysis (%)
Water (Positive control)	0.24	-
PBS (Negative control)	0.04	-
Native Pericardium	0.047	1.42
Decellularized Pericardium	0.050	3.14 ± 0.006
Composite Pericardium	0.046	1.07 ± 0.001

### 3.5 Kinetic Blood-Clotting Time Assay

**Figure 10:**
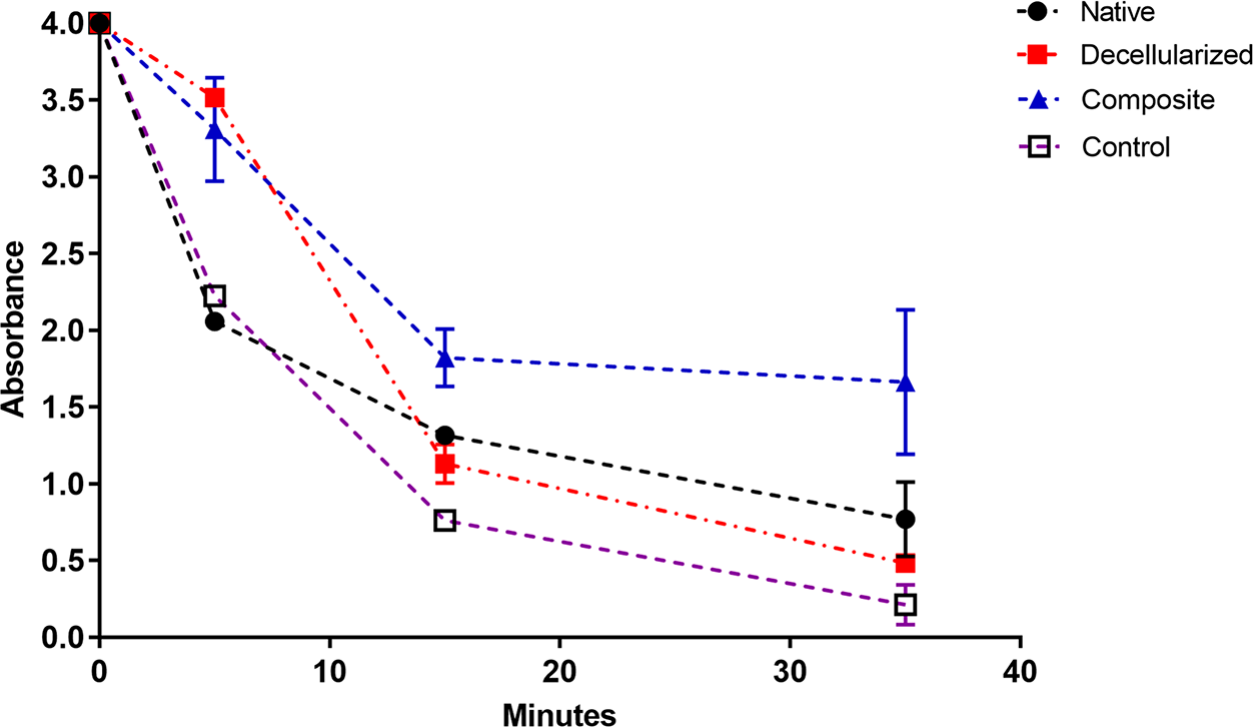
0th, 5th, 15th, and 35th minute blood-clotting kinetics graph of native, decellularized, and composite pericardium.

Whole blood-clotting kinetics were investigated by measuring the amount of free hemoglobin in terms of absorbance for native, decellularized, and composite pericardium (Figure 10).

The absorbance values of decellularized and composite pericardium were very close in the first 5 min, indicating that their coagulation kinetics were similar. However, after 15 min, a rapid decrease was observed in the level of free hemoglobin released from the blood interacting with the decellularized pericardium. The composite pericardium, on the other hand, demonstrated delayed coagulation kinetics having a higher level of released hemoglobin than the decellularized pericardium. By the 35th minute, almost all of the blood interacting with the decellularized pericardium was in the coagulated state. As for the native pericardium, even the amount of free hemoglobin after 5 min, is lower than the amount of free hemoglobin released from the composite pericardium at 35 min. This indicates that coagulation kinetics occur very rapidly in native pericardium. After 15 min, the native group followed the same coagulation kinetics as the decellularized group, and at the 35th minute, absorbance values of both groups were close.

### 3.6 In Vitro Cytotoxicity Assay

**Figure 11:**
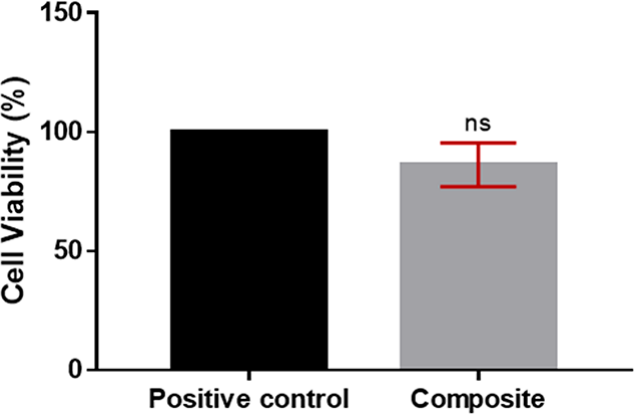
Cell viability (%) of the composite pericardium.

In order to determine the effects of carbon nanotubes on cell viability, cytotoxicity analysis was performed with the composite pericardium. In this assay, mitochondrial dehydrogenase enzymes in the living cells reduce the yellow tetrazolium dye MTT to its insoluble formazan, which has a purple color. The absorbance values (*n* = 3) obtained as the result of the MTT analysis were given in Figure 11. Cell viability was found to be 86.21% ± 7.51% in the composite pericardium. The positive control was accepted as 100%. Although there was a decrease in cell viability with carbon nanotube modification, the composite pericardium expressed no cytotoxic effect.

## 4 Discussion

Although thrombogenicity in bioprosthetic heart valves is a less common problem than in mechanical heart valves, it still poses a risk and eventually causes BHV failure. Bovine pericardium is the most widely used material in bioprosthetic heart valves because of the good hemodynamic profile, low degeneration, and low reoperation rate. However, collagen fibers on the surface of the pericardium are known to promote thrombogenicity.^18,30^ In the literature, it has been stated that deterioration of collagen structure by the decellularization process, changes in fiber morphologies and spaces, may lead to early thrombosis when used as a heart valve material.^31,32^

In this study, decellularized bovine pericardium was modified with carboxylated multiwalled carbon nanotubes to improve its thrombogenic properties and mechanical strength. The blood compatibility and antithrombogenic properties of carbon nanotubes have been studied with natural and synthetic polymers in the literature.^22−25^ Carbon nanotubes are also able to provide suitable mechanical properties for the heart valve material with their inert structure and high aspect ratio.

An SDS-based method was developed for pericardium decellularization and histological, biochemical, and morphological characterizations were performed. The amount of residual DNA remaining in the tissue was found to be 33,148 ± 5.51 ng after decellularization. This value remained below 50 ng, which is accepted as the limit value in the literature,^33^ and proved that the cells and residual DNA were removed successfully. ECM integrity was further characterized by quantitative analyses of extracellular matrix proteins such as collagen and sGAG. After decellularization, no significant change in the amount of collagen and sGAG was observed, indicating the success of the extracellular matrix preservation of decellularization protocol. As a matter of fact, a slight increase was observed in collagen content after decellularization, together with an increase in the area stained with blue in Masson’s Trichrome images, due to the exposure of more collagen fibers in the pericardium surface after the decellularization process. Although the collagen content was preserved or slightly decreased in studies in which SDS was not used in decellularization, a serious decrease in collagen content was found in studies using SDS, unlike our study. While the amount of sGAG in the decellularized pericardium was found to be an average of 50% in the literature, sGAG was preserved at a rate of 81% in this study.^17,34,35^

Composite pericardium was prepared by treatment of decellularized pericardium with 0.05% COOH–MWCNT solution. COOH–MWCNT concentration was determined according to the dispersion and forming homogeneous solution properties of COOH–MWCNTs in deionized water, and CNT toxicity studies in the literature.^36^ FTIR and TGA analyses were performed to determine the interaction efficiency of carbon nanotubes with the pericardial surface. In FTIR analysis, Amid 1, Amid 2, and Amid A peaks were found at the same absorbance in natural and decellularized pericardium.^28^ Due to the carboxylation of the carbon nanotube in the composite pericardium, the density increase at 3272.6 and 2918.5 cm^–1^ peaks and newly formed peaks at 2851.4, 1740, and 2360 cm^–1^ indicate −COOH CNTs on the pericardial surface.^27,37^ By thermogravimetric analysis, modification with carbon nanotubes was found to increase the thermal stability of the pericardium. The Native group demonstrated higher stability at first and second degradation temperatures, as it had intact extracellular matrix integrity and matrix components. However, after 600 °C, the composite pericardium showed higher thermal stability due to carbon nanotube modification. Significantly higher amounts of inorganic component remained in the composite at the end of heating compared to other groups. These results proved the efficient incorporation of carbon nanotubes on the pericardial surface.

After decellularization and modification with carbon nanotubes, surface morphologies were investigated in two and three dimensions by SEM and AFM. In both characterization methods, it was observed that the decellularization process reduced the roughness and heterogeneity on the surface, opened the layered structure, and created a flatter surface. It is known that the freeze–thawing procedure applied before SDS loosens the bonds in the tissues and disrupts cell membranes by forming ice crystals, so that the chemical and enzymatic agents applied afterward penetrate the tissue more effectively. The fact that the tissue has such a morphology after decellularization can be explained by this applied decellularization method.^38^

The binding efficiency and distribution of carbon nanotubes adhered to the surface in the composite pericardial group were also analyzed morphologically by SEM and AFM. Despite the heterogeneous surface structure of the pericardium and the different arrangement of collagen fibers, a homogeneous distribution of carbon nanotubes was observed on the surface, unlike carbon nanotube-decellularized composite tissue studies in the literature.^28^

Being able to provide appropriate mechanical properties is a significant issue when designing a leaflet for a heart valve. The lower/higher mechanical properties of the leaflets may affect the opening-closing rate and synchronization of the valves, leading to stenosis or regurgitation diseases over time, or may lead to early thrombogenesis due to blood flow. A slight decrease in tensile strength after decellularization process indicates that the applied decellularization protocol did not cause serious damage in the tissue. The decrease in elastic modulus after decellularization is an indication that the elastic elongation rate, that is, flexibility, increases with the decellularization of the tissue. These results are compatible with the macroscopic observations. Mechanical analysis results of the native and decellularized pericardium are in the same range as the bovine pericardial studies in the literature. In our study, the tensile strengths of the native and decellularized pericardium were found to be 14.37 ± 1.38 MPa and 11.94 ± 0.44 MPa, respectively, indicating a 17% decrease after decellularization process. While Young’s modulus of the native pericardium was 54.13 ± 0.42 MPa, it decreased to 33.26 ± 3.36 MPa after decellularization by showing a 38% decrease. In the study of Stimamiglio et al., the tensile strength of native and decellularized bovine pericardium was found to be 12.97 ± 6.22 MPa and 8.49 ± 3.41 MPa, respectively. This result indicates a 34% decrease in tensile strength after the decellularization process, which is double the decrease compared to our findings. Young’s modulus of native and decellularized pericardium was found to be 83.07 ± 35.64 MPa and 55.68 ± 22.99 MPa, respectively, showing a decrease of 33% similar to our study. In another study, Iop et al. found the tensile strength of native and decellularized bovine pericardium as approximately 11 and 9 MPa, respectively. The decrease of approximately 18% after the decellularization process is compatible to our data. Young’s modulus values were found to be approximately 32 and 25 MPa, respectively, indicating a decrease of approximately 22%.^34,39^ It was observed that the tensile strength of the decellularized pericardium was increased by the incorporation of carbon nanotubes, and the tensile strength of the native pericardium was restored. The elastic modulus data obtained from the stress–strain curve revealed a decrease in the elastic modulus of the tissue after the decellularization process. The decrease in elastic modulus indicates an increase in the flexibility of the material. While carbon nanotube modification did not significantly change the obtained flexible structure of the decellularized matrix, it mostly contributed to the increase of the mechanical strength of the tissue.

One of the important parameters that affects platelet adhesion and activation on biomaterial surfaces is surface characteristics (wettability, electrostatic charges, roughness). It has been reported in the studies that hydrophobic surfaces inhibit platelet adhesion and activation. Low surface energy of hydrophobic surfaces negatively affects the interface energy between the material surface and the plasma and, hence, the adsorption of plasma proteins and blood cells.^40−42^ In the study, surface energy values obtained by contact angle measurements revealed that the energy increased after the decellularization process of the native pericardial tissue. This can be associated with the loosening of the structure and the emergence of hydrophilic collagen fibers due to the destructive effect of the decellularization protocol on the tissue. This result was also supported by collagen staining (Masson’s Trichrome). The surface energy on the surface decreased due to the increased C–C bonds on the pericardium surface as a result of modification with MWCNT. Since the carboxylation of MWCNTs decreases the C–C bonds and increases the −COOH bonds instead, it did not increase the hydrophobicity as much as pristine MWCNT, but still increased compared to native and decellularized tissue. Contact angle of the composite surface was found to be increased 3.5 times, by providing a hydrophobic and platelet inhibiting surface. Contact angle of native pericardium was found to be higher than decellularized pericardium, which made the decellularized tissue more hydrophilic and more prone to platelet adhesion. This result proved the impact of surface characteristics for thrombogenicity studies. Besides surface hydrophobicity, electrostatic charges affect the behavior of plasma proteins and platelets on the surface. Using the −COOH functional group to bind MWCNT with the collagen on the surface resulted in the formation of a negatively charged pericardial surface. It is known from the literature that the carboxyl group has an inhibiting effect on protein adsorption, platelet adhesion, and activation. The adsorption of serum proteins to the surface, which is the first stage of thrombus formation, generally occurs on positively charged surfaces, since serum proteins, such as fibrinogen, and platelets have a net negative charge at physiological pH.^43^ Thus, charge repulsion occurs between the negatively charged material surface and serum proteins/platelets, and their attachment to the surface can be prevented. As another point, modification of the pericardial surface with MWCNT reduces platelet adhesion by minimizing the number of contact points where platelets can attach to the surface. Our findings were supported by the studies evaluating the relationship between contact angle and platelet adhesion/activation in which materials with similar contact angles to the MWCNT–pericardium contact angle reduce platelet adhesion. In fact, higher platelet adhesion inhibition was achieved compared to other studies.^42,44^*In vitro* stability analysis applied by collagenase digestion revealed that decellularized pericardium tissue show a similar degradation ratio to the literature.^45^ Although the results revealed that composite pericardium showed a better degradation profile than the decellularized pericardium and similar to native pericardium, they did not show the efficient resistance as decellularized pericardial tissues treated with cross-linkers highlighted in literature studies. This result is a preliminary finding for possible *in vivo* studies.

Platelet adhesion testing is one of the most important thrombogenicity assessments in the literature. A high number of platelet adhesion was observed in the SEM images of both native and decellularized pericardial surfaces. High-scale (10 μm) images showed that platelets tended to aggregate and formed connections with other platelet aggregates indicating that the platelet activation started. Determination of the activation status of platelets is determined by their morphological structures. According to the degree of activation, platelets are divided into five groups: dendritic, dendritic spread, spread, fully spread, and nonviable.^46−48^ However, in this study, since the platelets were mostly aggregated, their activation status was determined by the fibrin connections between the aggregates rather than the platelet morphology.

After surface modification with carbon nanotubes, a decrease of nearly 100% was observed in the number of platelets adhered to the surface. In addition to the successful inhibition of platelet adhesion, the fact that a few platelets that adhered were in round morphology indicates the platelets were in the passive state. Platelet adhesion was also investigated by Calcein-AM staining apart from SEM analysis. Microscope images showed a high number of viable platelets in the native and decellularized pericardium, consistent with SEM images, while only a few platelet adhesions were observed on the composite pericardial surface. According to the SEM analysis and other staining images, our study has achieved a more successful result in inhibiting platelet adhesion compared to other materials known as antithrombogenic in the literature such as heparin, polyethylene glycol (PEG), silver nanoparticles, nitric oxide, and many polymers known with good blood compatibility properties such as polyurethane (PU), polyglycerol sebacate (PGS), poly(lactic-*co*-glycolic acid) (PLGA), and poly(ε-caprolactone) (PCL).^10,21,22,49−51^

Hemolysis analysis, which is one of the important analyses in blood compatibility and thrombogenicity studies, is the deterioration of the membrane integrity of red blood cells after interacting with the surface. As a result of this, hemoglobin is released into the blood and the amount released can be measured as absorbance. It is known that nanoparticles can affect the membrane integrity of red blood cells through mechanical damage or reactive oxygen species.^25^ In this study, the effects of carbon nanotubes on red blood cells were investigated, and their hemolytic ratios were calculated. Native and composite pericardium were found to have similar rates of hemolysis with 1.42% and 1.07% hemolytic index, respectively. Even though the difference is not very high, hemolysis was slightly reduced in the composite pericardium. Since the pericardium is a natural tissue of the body, it is understandable that the effect of damaging blood cells, so the hemolysis index is low. Although decellularized pericardium showed a higher hemolysis index than native and composite, all groups are below the limit of 5% for biomaterials, indicating the good blood compatibility properties of the materials. In the study of Sakthikumar et al., Zein nanofibers were modified with different concentrations of SWCNTs ranging 0.2–1 wt %, and it was found that by increasing the carbon nanotube concentration, the hemolysis index of the material increased from 2.5% to 4%. Although it is still below the hemolysis limit, at high concentrations the carbon nanotubes tend to aggregate. Therefore, preparation of a homogeneous carbon nanotube solution and homogeneous interaction with the surface are very unlikely. More importantly, high concentrations of carbon nanotubes cause toxic effects, so it is recommended to use at low concentrations.^23,52^

As another marker of thrombogenicity, kinetic-blood coagulation analysis was performed by measuring the level of free hemoglobin released from unclotted blood at certain time points. Higher optical density indicates better antithrombogenicity.^53^ It was observed that even though the clotting dynamics of native and decellularized pericardium differ in the first 15 min, they exhibited very similar coagulation kinetics with lower optical densities after that time point, while the composite pericardium had the highest optical density indicating that it has the best anticoagulation property among all. The reduction in free hemoglobin released from native and decellularized groups was found to be 75% and 81.25%, respectively, indicating significant rates of coagulation. The reduction in the composite pericardium in this time period, on the other hand, was 55%. Although the decrease in coagulation rate after MWCNT modification was not as much as required, the coagulation profile was improved and followed a more stable dynamic compared to the other two groups at the end.

In vitro cytotoxicity analysis for carbon nanotube incorporated pericardium indicated that the composite material had a viability percentage of 86.21% ± 7.51%, above the 70% viability limit determined in the literature;^54^ thus it was concluded that it did not show cytotoxic properties.

To the best our knowledge, this study is a first in the literature and presented an innovative approach in terms of surface modification of pericardial tissue with carbon nanotubes and evaluation of thrombogenicity/blood compatibility for the development of heart valve leaflet materials. To take this study forward, further investigations should be carried out to evaluate the effects of the hemodynamic environment on the MWCNTs, in terms of thrombogenicity, bonding strength between MWCNTs and pericardium, fatigue property of composite leaflet due to the opening-closing cycles, safety and calcification, which is another important problem along with the thrombogenicity.

## 5 Conclusion

In this study, a nanocomposite leaflet material was developed to prevent thrombogenicity that may occur in artificial heart valves. Decellularized bovine pericardium, which is widely used in the clinic, was modified with multiwalled carbon nanotubes, and the thrombogenicity and other blood compatibility properties of the nanocomposite pericardium were evaluated. Carbon nanotube modification demonstrated higher blood compatibility and almost 100% antithrombogenic properties. In addition, the mechanical strength that the decellularization process reduced was replaced back to the native pericardium. This developed composite material may provide a new approach in the development of heart valve leaflets.
